# Editorial: Public Health Promotion and Medical Education Reform

**DOI:** 10.3389/fpubh.2022.918962

**Published:** 2022-05-04

**Authors:** Jian Chen, Zhiyong Zhang

**Affiliations:** ^1^School of Basic Medical Sciences, Guilin Medical University, Guilin, China; ^2^School of Public Health, Guilin Medical University, Guilin, China

**Keywords:** public health, medical education reform, public health education, trends and challenges, local/regional medical schools

Due to population aging and growing awareness of health-related issues, the demand for health services is increasing (1, 2). On the other hand, the shortage of healthcare workers prevails in most health care occupations, as demand outstrips supply. This problem may be particularly acute in local healthcare institutions where healthcare resources are limited (3). To address the supply-demand imbalance, various reforms of education and training of healthcare workers are underway in many parts of the world. Otherwise, the medical field has come to recognize the critical role of public health in prevention and control of diseases. The availability of early interventions would effectively reduce preventable illness, minimize complications, and lower health care cost burdens, with a corresponding reduction in demand for health workers (4). Consequently, many medical schools are engaged in incorporating public health and epidemiology courses into existing curricula. In this editorial, we provide a platform for most recent advances in teaching and learning innovations, along with issues relating to public health. The Research Topic represents a collection of 17 original research articles, two review articles and one opinion article ranging from theory to applications in both fields of medical education and public health.

To create a self-motivated learning environment, there has been a shift from teacher-centered to student-centered learning. This student-centered learning approach engages students to shape their own learning, where the teachers act as guides, helpers and facilitators of learning (5). A key result of this shift is the formation of “blended learning” that implement a student-centered approach to learning by using Internet technology and multimedia tools (6). One popular format to realize blended learning is the flipped classroom (FC). Application of FC in higher medical education is becoming more and more accepted because of its advantages over traditional learning (7). Ji et al. implemented FC in the whole course of physiology, and investigated both short- and long-term effects of FC on students' learning outcomes. Students were asked to get familiar with the basic learning objectives through pre-class learning using reading materials (e.g., videos and courseware) from Chinese biggest MOOC platform “iCourse.” The pre-class self-learning helps students to free up classroom time for higher-level thinking, and gives them more opportunity to discuss and interact with others. FC not only increased students' learning effectiveness but positively affect their long-term learning outcomes, indicating that FC enable students to acquire the skills of lifelong learning. As for students' preferences for online materials, Xu et al. found that students in the FC had a significant preference for mini-video with clear knowledge focus, but quite a few students did express positive attitudes to complete videos that may better illustrate the connection between different knowledge items. Likewise, Wu et al. applied FC based on micro-video class in pharmacology courses. This learning model significantly improved students' enthusiasm for learning, learning efficiency, self-directed learning ability, and problem solving skills, thus leading to improved learning performance. It can be concluded that success of blended learning depends not only on the quality of the course and the virtual environment, but also on whether there is individualized instruction of each student. In addition to flipped classroom, educators also implemented other types of blended learning according to their unique characteristics. Wang D. et al. combined massive open online courses (MOOCs) with case based learning (CBL) in teaching pathophysiology in a local medical university. They hope that the open online courses could offset the lack of qualified teachers and instructional materials in local medical institutions. Both students and teachers had positive attitude toward blended learning because students' achievement and motivation was greatly enhanced.

As the COVID-19 outbreak continues to evolve, it has brought tremendous changes in our daily life. For example, face-to-face education has been damaged in many countries, especially at the early stage of the COVID-19 pandemic (8). In such a case, online education is adopted on a large scale around the world for the first time. As review by Su et al. existing online course platforms in China such as MOOCs, Rain Classroom, WeChat, Moodle, QQ, and DingTalk have been further developed and implemented into online teaching (9). Accordingly, educators have developed more effective pedagogies for online learning, by which students have greater access to online medical resources and they can study on their own pace (10). Additionally, students' multiple abilities were improved, such as self-learning, independent thinking, communication and collaboration. Unexpectedly, this online teaching mode is more popular in Chinese postgraduate medical education during the COVID-19 pandemic. Postgraduates become even more active in online teaching than in traditional teaching. They are willing to communicate with teachers online in a relaxed atmosphere, and there appears to be more interaction between teachers and students. Another noteworthy phenomenon is that postgraduates could get more feedback from the educator in an online course. So it is evident that the online education is beneficial for most medical students in spite of the educational backgrounds.

COVID-19 is global health emergency, and has had a major impact on international higher education, which is to some extent caused by strict international border closures and irregular international flights at high cost (11). Liang et al. reviewed the most common difficulties faced by medical education based on the outcome-based education (OBE) concept for Bachelor of Medicine and Bachelor of Surgery (MBBS) in Chinese regional medical schools, as well as the solutions to overcome these difficulties. In the OBE model, ability training is integrated into professional education and provide support for the training of advanced applied medical talents. After the COVID-19 outbreak, MBBS education suffers from shortage of experienced teachers with good English proficiency and integrated online teaching platforms, and students may not be able to participate in internships. In response to these difficulties, teachers in these regional schools are encouraged to improve their online teaching competencies, including foreign language proficiency and teaching skills. The blended learning mode is integrated into MBBS education, while school supervision is adopted as strategies for quality assurance in medical education.

Before the COVID-19 pandemic, online learning did not account for the major modality for medical education, while now many medical institutions have established and strengthened their online learning and training programs (12, 13). Naidoo et al. designed a distance learning (DL) framework in anatomy teaching using the exemplar of the Head and Neck (H&N) course. The process of developing the framework was guided by the Analyse, Design, Develop, Implement, and Evaluate (ADDIE) model. They demonstrate that the DL-framework is an efficient learning approach and positively received by the students. This framework also developed students' cognitive ability such as communication, problem solving, and critical thinking.

Subsequent to the COVID-19 outbreak, the importance of public health education in addressing the pandemic has been highlighted (14). Since the health science students are future health care providers, their proper education regarding healthy lifestyle is necessary. Alotaibi et al. proved that the utilization of an online interactive educational workshop promoted acquisition of knowledge relevant to healthy lifestyle promotion. Meanwhile, the COVID-19 pandemic has exposed the urgent need for well-educated and appropriately trained public health leaders and managers to cope with public health challenges (15). Then a Chinese medical school established a special educational program for doctors of public health—the Doctoral Training Program of Public Health-Crisis Management (Cai et al.). The program meets the need of advanced public health practitioners in national emergencies by infusing public health content into foundation and advanced social service courses. The Program's graduate students developed their theoretical and communication abilities required for the management of national emergencies. In addition, their experiences working with experts from Disease Control and Prevention (CDC) greatly improved the practical and leadership-related capacity of public health crisis management. It may support the development of advanced public health policy and health care education in China. The COVID-19 crisis also poses unique challenges for clinical training. With the aim of developing effective and sound training models for epidemic prevention and control, a conceive-design-implement-operate (CDIO) mode was adopted to improve the theoretical knowledge and practical skills of obstetrics and gynecology (O&G) residents in COVID-19 epidemic prevention protocols (Wang X. et al.). This timely training is beneficial in alleviating work pressure and improving professional identity of residents.

More importantly, the COVID-19 has pried our eyes open to the importance of public health. The primary prevention interventions lead to reduction in incidence of diseases, and a healthier population would have lower health care spending (16). As is known, the nation's future health will be greatly determined by the health status and future health risk among children and young people (17). Peng et al. found that the overweight of teenagers was positively related to the proportion of biochemical abnormalities in their blood, while the healthy behavior helped to improve biochemical indexes and control overweight, and from here it is necessary to bring health education into the national education system. And a school health education program should be established to let teenagers take physical exercise as a way of life. However, in many developing countries, there is the lack of health education, which may lead to severe public health problems. It was found that Chinese college students in Henan had preliminary understanding of HIV knowledge, but they did not know enough about sexual transmission of HIV (Zhang et al.). Notably, the college students' attitude toward HIV-infected patients was generally negative. This may be alleviated by raising awareness of AIDS health education knowledge. That is, the adequate health knowledge can create positive attitude toward health care. Similarly, a cross sectional community-based study about blood donation in India demonstrated that most participants had favorable attitudes toward blood donation, among who most were knowledgeable (Samreen et al.).

The WHO defines health as a state of complete physical, mental, and social wellbeing and not merely the absence of disease and infirmity (18). Mental health is also an integral component of general health. Yet, mental health is neglected more as compared to physical health. Although many national governments have paid increasingly close attention to the field of mental health, the COVID-19 pandemic also had enormous impact on mental health (19). Individuals affected in the pandemic may have many mental health problems such as depression, anxiety, stress, panic attack, and sleep disorders (20). Therefore, it is essential to provide mental health services to the public, whereas access to mental health services should be increased. In this case, Mat Ruzlin et al. held a campaign called “Mind your Mental Health Carnival”—a hybrid carnival which consists of in-person and virtual attendants. They delivered mental health content and health interventions using web-based platforms. As a result, this hybrid approach that combined face-to-face interactions and virtual learning improved mental health literacy among participants. Meanwhile, free online therapy sessions were offered to identify highly vulnerable individuals during the screening process. The study proved the feasibility of this digital approach in providing mental health support and treatment during the COVID-19 pandemic.

Another public health issue is how to effectively disseminate health knowledge to the public. Given the fact that much people are willing to seek out information and interact with others via social media, most medical institutions have run an official account for professional health knowledge dissemination (21). Through analyzing readers experiences of reading articles published by the official WeChat account of a hospital in China, Bian et al. demonstrated that the modern science and technology made it much easier for people to learn about the health information of various diseases, contributing to effective prevention and treatment of chronic diseases. Hence, healthcare workers should pay more attention to the use of social media so as to popularize the concept of health. There is no doubt that physicians are responsible for dissemination of health information and education. Regardless of education level, the patients were more likely to report attempted behavior change according physician advice for lifestyle change, while compliance with advice was affected by health education on physical activity (Chen et al.).

When it comes to public health, we can not ignore the effects of economic issues on health care. Economic conditions would influence personal health, health care services and population health. At personal level, the costs of medical services has been identified to be a large factor in patients' decision-making (22). On the other hand, the consumption of drugs, a major source of medical cost, is on the rapid rise with advanced healthcare (23). Although the therapeutic effectiveness of generic drugs are similar to their brands, the attitudes of practicing pharmacists and pharmacy students are ambivalent due to the different knowledge level regarding generic medicines. The better awareness of the generic medicines, the more liable the practicing pharmacists and pharmacy students have positive attitudes to generic drugs (Al-Arifi et al.). As future pharmacist, pharmacy students need to grasp adequate information regarding generic medicines. More severely, economic conditions influences personal protection of frontline workers in the COVID-19 pandemic. Compared with general population, frontline workers have a higher risk of COVID-19 virus infection, and thus personal protective equipment is widely used to avoid virus infection (24). Nevertheless, elevated cost negatively influences workers' intention to use personal protective equipment, though they have adequate knowledge about this pandemic and possess a greater propensity to use the equipment (Irfan et al.).

Medical students, as special frontline healthcare workers, seems to be more susceptible to the virus because of the lack of skills and experience in managing emergencies. Feng et al. focused on the performance of medical post-graduates during the COVID-19 pandemic. Indeed, this pandemic had an impact on student clinical internships, and they became more aware of personal protection. Students took different protective measures against COVID-19 infection, but they still worry about the insufficient personal protective measures and the possibility of cross-infection in the hospital.

Overall, the articles outlined above in this Research Topic provide insights into better understanding of medical education reform and public health, and analyses the impact of COVID-19 pandemic on the education and training of healthcare workers and public health activity. This global emergency would lead to improved healthcare delivery and health systems development.

## Author Contributions

All authors contributed to the article and approved it for publication.

## Funding

This research was supported by the National Natural Science Foundation of China (Grant Nos. 81973574 and 82174082) and the Natural Science Foundation of Guangxi (Grant No. 2019GXNSFFA245001).

## Conflict of Interest

The authors declare that the research was conducted in the absence of any commercial or financial relationships that could be construed as a potential conflict of interest.

## Publisher's Note

All claims expressed in this article are solely those of the authors and do not necessarily represent those of their affiliated organizations, or those of the publisher, the editors and the reviewers. Any product that may be evaluated in this article, or claim that may be made by its manufacturer, is not guaranteed or endorsed by the publisher.
